# Dr. Thomas Crawford Hayes

**Published:** 1909-04-24

**Authors:** 


Dr. Thomas Crawford Hayes, the well-known gynæcologist, died on Monday at Clarges Street, Mayfair. Dr. Hayes was formerly a senior moderator and gold medallist at Trinity College, Dublin. In 1872 he obtained the membership of the Royal College of Physicians of London, and in 1875 obtained the M.D. Dublin, whilst in 1889 he became a Fellow of the Royal College of Physicians. Dr. Hayes was emeritus professor of obstetric medicine at King's College and consulting physician for diseases of women to King's College and Royal Free Hospitals. He had also held appointments at the Evelina Hospital for Sick Children and the General Lying-in Hospital, and was formerly a member of the English Conjoint Examining Board.

